# Activation of Both CB1 and CB2 Endocannabinoid Receptors Is Critical for Masculinization of the Developing Medial Amygdala and Juvenile Social Play Behavior

**DOI:** 10.1523/ENEURO.0344-16.2017

**Published:** 2017-01-27

**Authors:** Kathryn J Argue, Jonathan W VanRyzin, David J Falvo, Allison R Whitaker, Stacey J Yu, Margaret M McCarthy

**Affiliations:** Department of Pharmacology, University of Maryland School of Medicine, Baltimore, MD 21201

**Keywords:** amygdala, development, endocannabinoid, juvenile play behavior, neuronal morphology, rat

## Abstract

Juvenile social play behavior is a shared trait across a wide variety of mammalian species. When play is characterized by the frequency or duration of physical contact, males usually display more play relative to females. The endocannabinoid system contributes to the development of the sex difference in social play behavior in rats. Treating newborn pups with a nonspecific endocannabinoid agonist, WIN55,212-2, masculinizes subsequent juvenile rough-and-tumble play behavior by females. Here we use specific drugs to target signaling through either the CB1 or CB2 endocannabinoid receptor (CB1R or CB2R) to determine which modulates the development of sex differences in play. Our data reveal that signaling through both CB1R and CB2R must be altered neonatally to modify development of neural circuitry regulating sex differences in play. Neonatal co-agonism of CB1R and CB2R masculinized play by females, whereas co-antagonism of these receptors feminized rates of male play. Because of a known role for the medial amygdala in the sexual differentiation of play, we reconstructed Golgi-impregnated neurons in the juvenile medial amygdala and used factor analysis to identify morphological parameters that were sexually differentiated and responsive to dual agonism of CB1R and CB2R during the early postnatal period. Our results suggest that sex differences in the medial amygdala are modulated by the endocannabinoid system during early development. Sex differences in play behavior are loosely correlated with differences in neuronal morphology.

## Significance Statement

Juvenile social play behavior is a critical component for proper brain development and the acquisition of social competence in the majority of mammalian species. In juvenile rats, males exhibit higher numbers of rough-and-tumble play events relative to females. This difference in rat play behavior is programmed by the actions of steroid hormones during the early postnatal sensitive period for sexual differentiation of the brain. Here we demonstrate a requirement for combined activation or inhibition of both endocannabinoid receptors for masculinization or feminization of the neural circuitry for play, respectively. Furthermore, our findings suggest a correlation between playfulness and neuronal morphology in the medial amygdala.

## Introduction

Conspecific social play is a critical component of the juvenile period for a wide variety of mammalian species (Gruendel and Arnold, 1969; Byers and Walker, 1995; Arthur et al., 1999; van den Berg et al., 1999; Spinka et al., 2001; Graham and Burghardt, 2010; Pellis et al., 2010; Siviy, 2016). Deficits in social play, including delayed onset, decreased intensity, problems responding to social cues, and withdrawal from social play situations leading to isolation, are observed in children diagnosed with psychiatric disorders, such as autism spectrum disorder, early-onset schizophrenia, and attention deficit hyperactivity disorder (Gruendel and Arnold, 1969; Byers and Walker, 1995; Arthur et al., 1999; van den Berg et al., 1999; Møller and Husby, 2000; Spinka et al., 2001; Jordan, 2003; Strous et al., 2004; Cordier et al., 2010; Graham and Burghardt, 2010; Pellis et al., 2010). Identifying variables contributing to individual playfulness and that produce a more desirable play partner will help elucidate factors critical for normal social development as well as how those factors are altered during aberrant development.

A common and trans-species form of social behavior is rough-and-tumble play, or play fighting (Henry and Herrero, 1974; Pellis and Pellis, 1997, 1998; Cordoni, 2009). In juvenile rats, rough-and-tumble play consists of a complex set of behaviors that require appropriate initiation and responses to play solicitation (Pellis et al., 1997; Himmler et al., 2014; Argue and McCarthy, 2015a). By postnatal day 20 (PN20), juvenile rats display the full repertoire of rough-and-tumble play behaviors, including initiation of play (pouncing) and responses to play solicitation (pinning; Pellis et al., 1997; Himmler et al., 2014; Argue and McCarthy, 2015a). The majority of species exhibit a sex difference in rough-and-tumble play, with males having increased frequency and duration relative to females (Leresche, 1976; Olioff and Stewart, 1978; Takahashi and Lore, 1983; Thor and Holloway, 1985, 1986; Watson and Croft, 1993; Pellis, 2002; Palagi et al., 2007; Parent and Meaney, 2008; Auger and Olesen, 2009; Krebs-Kraft et al., 2010; Argue and McCarthy, 2015a). However, there are some discrepancies in the literature regarding sex differences in rough-and-tumble play. When observations are made outside of a controlled environment, it is often difficult to determine how specific conditions of the study could have contributed to the findings. For example, in very young Gelada baboons, the sex with the greatest number of play bouts, defined by instances of play biting, mouthing, slapping, jumping at, tail pulling, object play, boxing, chasing, wrestling, and other nonspecified forms of rough-and-tumble play, varies depending on the month studied, with females playing more than males in the majority of months. However when animals of an older age cohort are observed, males consistently play more frequently (Barrett et al., 1992). In baboons, there are seasonal changes in levels of play in younger animals, with females playing more than males in the majority of months, and in older animals no sex difference is observed (Cheney 1978). This is just one example of many illustrating how different definitions of what constitutes social play, the age of the animals studied, and the specific conditions under which the animals are studied can impact the observation of sex differences in play. Sex differences in juvenile rough-and-tumble play of the popular laboratory species, *Rattus norvegicus*, have been found in the frequency of pouncing (play initiation), the frequency of pinning (playful defense), and the amount of time engaged in play. These observations can differ depending on the rat strain and methodology (i.e., whether play was assessed in a group or pairs and whether they consisted of same- or mixed-sex individuals; Argue and McCarthy, 2015b).

Execution of play behavior involves multiple brain regions. Meaney and McEwen (1986) determined that testosterone acting in the neonatal medial amygdala is sufficient to masculinize juvenile play behavior. In this same brain region, there is a developmental sex difference in the endocannabinoid system (Krebs-Kraft et al., 2010) which comprises two principal ligands, 2-arachidonoyl glycerol (2-AG) and anandamide (AEA), and two key receptors, CB1R and CB2R (Lu and Mackie, 2015). The developing male amygdala has higher levels of 2-AG and to a lesser extent, AEA, and pharmacologically increasing the endocannabinoid signal in neonatal females via administration of the nonspecific endocannabinoid agonist, WIN55,212-2, increases juvenile rough-and-tumble play behavior to the levels observed in males (Krebs-Kraft et al., 2010). To further explore which endocannabinoid receptors transduce the endocannabinoid signal to modify development of the social play circuitry, we here use specific CB1R and CB2R agonists or antagonists and explore the impact of single or combined treatment.

## Materials and Methods

### Animals

Sprague-Dawley rats (Harlan) mated in our facility or ordered as pregnant dams were allowed to deliver normally under standard laboratory conditions (total *n* = 160 pups from 16 litters). On the day of birth (PN0) pups were given paw tattoos to identify treatment groups. Pups were weaned on PN22 and housed in pairs or groups consisting of no more than three individuals of the same sex in polycarbonate cages (20 × 40 × 20 cm) with corncob bedding under a reverse 12:12 h light/dark cycle. Food and water were available ad libitum. All breeding and experimental procedures were approved by the Institutional Care and Use Committee at the University of Maryland, Baltimore and performed in accordance with national animal care and use guidelines.

### Play experiment 1: effect of CB1R or CB2R specific agonists or antagonists on play behavior

Pups were given daily intraperitoneal (i.p.) injections for four consecutive days (PN0–3). Females received 1 mg/kg ACEA (a CB1-specific agonist; Tocris), 1 mg/kg GP1a (a CB2-specific agonist; Tocris), or vehicle (saline with 2% ethanol). Males received 1 mg/kg AM281 (a CB1-specific antagonist/inverse agonist; Tocris), 1 mg/kg AM630 (a CB2-specific antagonist/inverse agonist; Tocris), or vehicle (saline with 2% ethanol and 2% DMSO; *n* = 6–9 individuals from each treatment group). The injection site was sealed with VetBond (3M) to prevent leakage. On PN27, animals were tested for 10 min in a 96 × 80 × 41-cm open field with a 16-cm^2^ grid to rule out potential confounds that differences in activity or anxiety-like behavior might have on play. Behavior was videorecorded and scored to determine levels of locomotion (indicated by the number of gridlines crossed) or anxiety-like behavior (time spent in the center zone). On PN28–37, juvenile social play was assessed in groups of six consisting of noncagemates from each of the different treatment groups (female vehicle, male vehicle, female ACEA, female GP1a, male AM281, and male AM630). Animals were marked with a marker for identification and placed in a 49 × 37 × 24-cm enclosure with TEK-Fresh cellulose bedding (Harland Laboratories), allowed to acclimate for 2 min, and videorecorded for 10 min. All social play behavior occurred under red-light illumination during the dark phase of the cycle. The videos were scored to determine the number of times pouncing, pinning, or boxing behaviors occurred.

### Play experiment 2: effects of CB1 and CB2 receptor co-agonism on play behavior

Pups were given daily i.p. injections (PN0–3) as described in Experiment 1. Male and female pups received 1 mg/kg ACEA + 1 mg/kg GP1a, 1 mg/kg WIN55,212-2 (Tocris), or vehicle (saline with 4% ethanol; *n* = 6–9 individuals from each treatment group). On PN27, animals were tested in the open field, and for 8 days (PN28–37), social play was assessed as described for Experiment 1. Animals were placed in groups of six consisting of noncagemates from each of the treatment groups (female vehicle, male vehicle, female ACEA+GP1a, female WIN, male ACEA+GP1a, male WIN).

### Play experiment 3: effects of CB1 and CB2 receptor co-antagonism on play behavior

Pups were given daily i.p. injections (PN0–3) as described for Experiment 1. Male and female pups received 1 mg/kg AM281 + 1 mg/kg AM630 or vehicle (saline with 4% DMSO; *n* = 7–10 individuals from each treatment group). On PN26, animals were tested in the open field, and on PN27–34, social play behavior was assessed as described for Experiment 1. For this experiment, animals were placed in same-sex/treatment pairs with a noncagemate partner rather than in a mixed-treatment/sex group. Pairing animals of the same sex and treatment and hence similar levels of playfulness helps to eliminate any effects that the play partners can have on an individual’s play, such as a more playful partner increasing play in a contagious manner or a less playful partner reducing play through negative reinforcement. These effects of play partners are exemplified by the observations that male-male dyads will play more than mixed-sex pairs, whereas female-female dyads will play less than both male-male and mixed-sex pairs. This paired paradigm also increases the total number of play events observed for an individual relative to what is observed with a group paradigm.

### 3D neuronal reconstruction

**Fig. 1.**
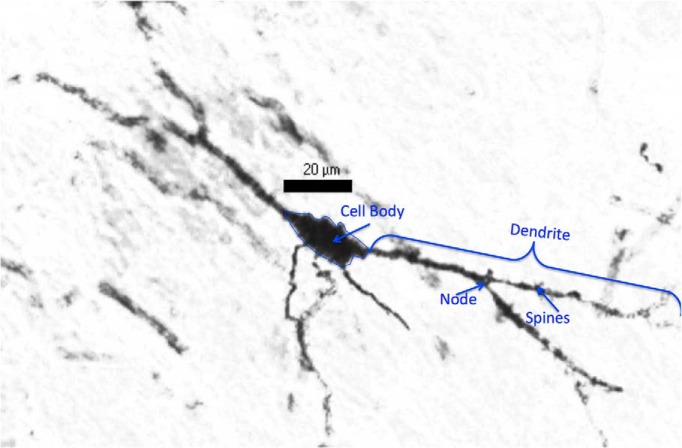
Representative image of a PN26 Golgi-impregnated neuron. A Golgi-impregnated neuron was imaged at 40× magnification. The cell body is outlined, and a dendrite, node, and spine are indicated to illustrate the parameters of neuronal morphology that were included in our analysis.

To reconstruct neurons on PN4, pups were anesthetized with FatalPlus and perfused intracardially with 0.9% saline (*n* = 10 animals of each sex). Brains were removed and placed into Golgi solution (1:1 solution of 5% potassium dichromate and 5% mercuric chloride that was added to 5% potassium chromate in a 2:5 ratio) and left for 48 h, after which the brains were switched to fresh Golgi solution where they remained for 8 d. Brains were removed from Golgi solution and incubated in 30% sucrose for 4 d, followed by incubation in Solution C (from Cedarlane Labs FD Ropid GolgiStain Kit) for 4 d. Brains were coronally sectioned on a cryostat at a thickness of 100 μm and mounted onto gelatin-subbed slides. Slides were developed with incubation in a 30% ammonium hydroxide solution, then 1% Dektol (Kodak) solution, and finally 18% Fix (Kodak) solution, counterstained with 0.2% methylene blue, cleared with ascending alcohol, defatted with xylenes, and coverslipped with DPX mounting medium. Six neurons from ≥3 different sections were reconstructed from the right and left hemisphere of each individual. Slides were numerically coded, blinding the tracer to the sex of each individual. Well-impregnated neurons were selected based on the presence of a filled cell body and dendrites that were filled to their ends. Because some cracking of the tissue did occur with the development protocol, only neurons that came to a natural end before reaching a crack were analyzed. These well-impregnated neurons were reconstructed under a 100× oil-immersion objective using Neurolucida (Microbrightfield) interfaced with a Nikon Eclipse E600 microscope and an MBF Bioscience CX9000 Digital Camera. Cell body area, number of primary dendrites, number of nodes (branch points on a dendritic process), spine density (total number of spines on each neuron divided by total dendritic length), total dendritic length, and average dendritic length (total dendritic length divided by number of primary dendrites) were quantified and compared (Fig. 1).

For the reconstruction of neurons on PN26, brains were taken as described above from behaviorally naive animals that were treated (PN0–3) with 1 mg/kg ACEA, 1 mg/kg GP1a or both, or vehicle (saline with 4% ethanol), via i.p. injection (*n* = 3–4 animals from each treatment group). Brains were impregnated, cut, stained, and reconstructed as described above except that they were incubated in Golgi solution for 12 d after the switch to fresh solution at 48 h postmortem. As done at PN4, six neurons from each hemisphere from each individual were reconstructed.

### Statistical analysis

Data analysis was performed using SPSS for Windows (IBM), GraphPad Prism, and R (version 3.2.1). All data sets were tested for normal distribution using a Kolmogorov–Smirnov test.

The total number of play events (total number of pounces, pins, and boxing events) was combined from all days of analysis. Outliers were removed using the ROUT method with Q = 1%, and data were analyzed using either ANOVA or two-way ANOVA, as indicated for each experiment. Play was also split into its two major components, initiation of play (indicated by the number of pounces) and response to play initiation (indicated by the number of pins). Because of the infrequency of boxing events, this parameter was not analyzed independently. Pouncing and pinning were analyzed using either ANOVA or a two-way ANOVA, as indicated for each experiment. If a significant ANOVA or two-way ANOVA was obtained for frequency of play, pouncing, or pinning, *t*-tests with a Welch correction were performed to test *a priori* hypotheses. Open-field behavior (gridlines crossed and centering time) was analyzed by ANOVA or two-way ANOVA as indicated for each experiment.

Neuronal morphology at PN4 was analyzed by *t*-tests with a Welch correction. Parameters of neuronal morphology at PN26 were analyzed using factor analyses. The data were log transformed, and the factor analyses were performed with varimax rotation, which produces orthogonal values and preserves the variance of the original data. The resulting factors were retained if their eigenvalue was >1 (based on the Kaiser criterion), and a 0.3 significance cutoff was used to determine loading of a parameter into a factor. Individual factor scores were calculated for each cell and averaged with others from the same experimental groups. These data were analyzed using ANOVA or *t*-tests with a Welch correction.

For all analyses, differences were considered significant when *p* < 0.05. All data are depicted as mean with SEM. The results for all analyses are shown in Table 1. Superscript letters listed with *p*-values correspond to the statistical tests shown in Table 1.

**Table 1.** Summary of statistical analysis.

Line	Data analyzed	Results	Data structure	Type of test	Observed power or confidence interval
a	Total play: female vehicle, female ACEA, female GP1a	*F*(2, 224) = 0.28, *p* = 0.756	Normal	ANOVA	0.094
b	Pouncing: female vehicle, female ACEA, female GP1a	*F*(2, 222) = 0.2.713, *p* = 0.069	Normal	ANOVA	0.533
c	Pinning: female vehicle, female ACEA, female GP1a	*F*(2, 210) = 0.699, *p* = 0.498	Normal	ANOVA	0.167
d	Number of gridlines crossed: female vehicle, female ACEA, female GP1a	*F*(2, 23) = 1.008, *p* = 0.383	Normal	ANOVA	0.201
e	Time spent in the center zone: female vehicle, female ACEA, female GP1a	*F*(2, 23) = 0.262, *p* = 0.772	Normal	ANOVA	0.086
f	Total play: male vehicle, male AM281, male AM630	*F*(2, 208) = 1.314, *p* = 0.271	Normal	ANOVA	0.282
g	Pouncing: male vehicle, male AM281, male AM630	*F*(2, 208) = 2.103, *p* = 0.125	Normal	ANOVA	0.429
h	Pinning: male vehicle, male AM281, male AM630	*F*(2, 187) = 5.483, *p* = 0.005	Normal	ANOVA	0.845
i	Pinning: male vehicle, male AM281	*p* = 0.675		Tukey *post hoc*	–0.4573 to 0.2162
j	Pinning: male vehicle, male AM630	*p* = 0.004		Tukey *post hoc*	–0.7868 to –0.1227
k	Number of gridlines crossed: male vehicle, male AM281, male AM630	*F*(2, 21) = 1.150, *p* = 0.339	Normal	ANOVA	0.221
l	Time spent in the center zone: male vehicle, male AM281, male AM630	*F*(2, 21) = 1.568, *p* = 0.236	Normal	ANOVA	0.289
m	Total play: male vehicle, female vehicle, male WIN, female WIN, male ACEA+GP1a, female ACEA+GP1a	*F*(2, 360) = 4.449, *p* = 0.012	Normal	2-Way ANOVA: sex × treatment interaction	0.762
n		*F*(1, 360) = 4.108, *p* = 0.043		Main effect of sex	0.524
0	*F*(1, 360) = 2.275, *p* = 0.104	Main effect of treatment		0.461	
p	Total play: male vehicle, female vehicle	*t*(109.2) = 2.666, *p* = 0.0089		*t*-test	0.5331 to 3.624
q	Total play: female vehicle, female WIN	*t*(127.3) = 2.01, *p* = 0.0465		*t*-test	0.023 to 2.919
r	Total play: female vehicle, female ACEA+GP1a	*t*(114.7) = 2.071, *p* = 0.0406		*t*-test	0.07583 to 3.415
s	Total play: male vehicle, female WIN	*t*(112.5) = 0.7378, *p* = 0.4622		*t*-test	–1.024 to 2.24
*t*	Total play: male vehicle, female ACEA+GP1a	*t*(116.8) = 0.3607, *p* = 0.719		*t*-test	–1.497 to 2.163
u	Total play: male vehicle, male WIN	*t*(83.32) = 0.882, *p* = 0.3803		*t*-test	–1.182 to 3.066
v	Total play: male vehicle, male ACEA+GP1a	*t*(109) = 1.711, *p* = 0.0899		*t*-test	–3.2 to 0.2348
w	Pouncing: male vehicle, female vehicle, male WIN, female WIN, male ACEA+GP1a, female ACEA+GP1a	*F*(2, 359) = 6.914, *p* = 0.001	Normal	2-Way ANOVA: sex × treatment interaction	0.923
×		*F*(1, 359) = 3.474, *p* = 0.63		Main effect of sex	0.46
y		*F*(2, 359) = 3.7, *p* = 0.026		Main effect of treatment	0.677
z	Pouncing: male vehicle, female vehicle	*t*(118.7) = 2.347, *p* = 0.0206		*t*-test	0.2161 to 2.551
aa	Pouncing: female vehicle, female WIN	*t*(131) = 1.358, *p* = 0.1767		*t*-test	–0.3653 to 1.966
bb	Pouncing: female vehicle, female ACEA+GP1a	*t*(119.8) = 2.123, *p* = 0.0358		*t*-test	0.09501 to 2.724
cc	Pouncing: male vehicle, female WIN	*t*(116.3) = 0.947, *p* = 0.3456		*t*-test	–0.6366 to 1.803
dd	Pouncing: male vehicle, female ACEA+GP1a	*t*(115.3) = 0.038, *p* = 0.9698		*t*-test	–1.388 to 1.336
ee	Pouncing: male vehicle, male WIN	*t*(77.85) = 1.917, *p* = 0.0589		*t*-test	–0.06252 to 3.323
ff	Pouncing: male vehicle, male ACEA+GP1a	*t*(108.9) = 2.069, *p* = 0.0409		*t*-test	–2.554 to –0.05488
gg	Pinning: male vehicle, female vehicle, male WIN, female WIN, male ACEA+GP1a, female ACEA+GP1a	*F*(2, 329) = 3.171, *p* = 0.043	Normal	2-Way ANOVA: sex × treatment interaction	0.605
hh		*F*(1, 329) = 4.158, *p* = 0.042		Main effect of sex	0.529
ii		*F*(2, 329) = 3.53, *p* = 0.03		Main effect of treatment	0.655
jj	Pinning: male vehicle, female vehicle	*t*(70.79) = 3.206, *p* = 0.002		*t*-test	0.2814 to 1.207
kk	Pinning: female vehicle, female WIN	*t*(99.25) = 5.11, *p* < 0.0001		*t*-test	0.5536 to 1.256
ll	Pinning: female vehicle, female ACEA+GP1a	*t*(89.6) = 2.911, *p* = 0.0045		*t*-test	0.1803 to 0.9554
mm	Pinning: male vehicle, female WIN	*t*(96.71) = 0.6144, *p* = 0.5404		*t*-test	–0.6797 to 0.3584
nn	Pinning: male vehicle, female ACEA+GP1a	*t*(103.2) = 0.6438, *p* = 0.5211		*t*-test	–0.367 to 0.7198
oo	Pinning: male vehicle, male WIN	*t*(94.97) = 0.002952, *p* = 0.9977		*t*-test	–0.576 to 0.5777
pp	Pinning: male vehicle, male ACEA+GP1a	*t*(105) = 0.4127, *p* = 0.6807		*t*-test	–0.4773 to 0.7281
qq	Number of gridlines crossed: male vehicle, female vehicle, male WIN, female WIN, male ACEA+GP1a, female ACEA+GP1a	*F*(2, 45) = 2.497, *p* = 0.095	Normal	2-Way ANOVA: sex × treatment interaction	0.471
rr		*F*(2, 45) = 0.39, *p* = 0.536		Main effect of sex	0.094
ss		*F*(1, 45) = 1.145, *p* = 0.329		Main effect of treatment	0.237
tt	Time spent in the center zone: male vehicle, female vehicle, male WIN, female WIN, male ACEA+GP1a, female ACEA+GP1a	*F*(2, 45) = 0.701, *p* = 0.502	Normal	2-Way ANOVA: sex × treatment interaction	0.16
uu		*F*(1, 45) = 1.592, *p* = 0.215		Main effect of sex	0.234
vv		*F*(2, 45) = 0.936, *p* = 0.401		Main effect of treatment	0.2
ww	Total play: male vehicle, female vehicle, male AM281+AM630, female AM281+AM630	*F*(1,240) = 7.081, *p* = 0.008	Normal	2-Way ANOVA: sex × treatment interaction	0.755
xx		*F*(1, 240) = 11.592, *p* = 0.001		Main effect of sex	0.924
yy		*F*(1, 240) = 5.207, *p* = 0.023		Main effect of treatment	0.623
zz	Total play: male vehicle, female vehicle	*t*(126) = 4.011, *p* = 0.0001		*t*-test	–9.707 to –3.293
aaa	Total play: male vehicle, male AM281+AM630	*t*(105.5) = 3.195, *p* = 0.0018		*t*-test	–8.584 to –2.01
bbb	Total play: female vehicle, male AM281+AM630	*t*(106.1) = 0.7202, *p* = 0.473		*t*-test	–4.515 to 2.109
ccc	Total play: female vehicle, female AM281+AM630	*t*(106.2) = 0.2976, *p* = 7.666		*t*-test	–2.3 to 3.113
ddd	Pouncing: male vehicle, female vehicle, male AM281+AM630, female AM281+AM630	*F*(1, 240) = 0.7072, *p* = 0.008	Normal	2-Way ANOVA: sex × treatment interaction	0.754
eee		*F*(1, 240) = 13.742, *p* < 0.0001		Main effect of sex	0.958
fff		*F*(1, 240) = 6.151, *p* = 0.014		Main effect of treatment	0.695
ggg	Pouncing: male vehicle, female vehicle	*t*(124,9) = 4.191, *p* < 0.0001		*t*-test	–7.361 to –2.639
hhh	Pouncing: male vehicle, male AM281+AM630	*t*(107.5) = 3.225, *p* = 0.0017		*t*-test	–4.036 to 1.252
iii	Pouncing: female vehicle, male AM281+AM630	*t*(103,6) = 0.8051, *p* = 0.4226		*t*-test	–3.337 to 1.41
jjj	Pouncing: female vehicle, female AM281+AM630	*t*(107.8) = 0.1472, *p* = 0.8833		*t*-test	–1.754 to 2.035
kkk	Pinning: male vehicle, female vehicle, male AM281+AM630, female AM281+AM630	*F*(1, 235) = 10.073, *p* = 0.002	Normal	2-Way ANOVA: sex × treatment interaction	0.885
lll		*F*(1, 235) = 1.538, *p* = 0.216		Main effect of sex	0.235
mmm		*F*(1, 235) = 2.716, *p* = 0.101		Main effect of treatment	0.375
nnn	Pinning: male vehicle, female vehicle	*t*(122.1) = 3.015, *p* = 0.0031		*t*-test	–2.532 to –0.5248
ooo	Pinning: male vehicle, male AM281+AM630	*t*(106.5) = 3.251, *p* = 0.0015		*t*-test	–2.687 to –0.6514
ppp	Pinning: female vehicle, male AM281+AM630	*t*(101.2) = 0.2961, *p* = 0.7678		*t*-test	–0.8049 to 1.087
qqq	Pinning: female vehicle, female AM281+AM630	*t*(121.8) = 1.189, *p* = 0.2368		*t*-test	–0.3514 to 1.408
rrr	Number of gridlines crossed: male vehicle, female vehicle, male AM281+AM630, female AM281+AM630	*F*(1, 32) = 0.119, *p* = 0.733	Normal	2-Way ANOVA: sex × treatment interaction	0.063
sss		*F*(1, 32) = 0.001, *p* = 0.872		Main effect of sex	0.05
ttt		*F*(1, 32) = 0.071, *p* = 0.792		Main effect of treatment	0.058
uuu	Time spent in the center zone: male vehicle, female vehicle, male AM281+AM630, female AM281+AM630	*F*(1, 32) = 0.377, *p* - 0.544	Normal	2-Way ANOVA: sex × treatment interaction	0.091
vvv		*F*(1, 32) = 0.008, *p* = 0.93		Main effect of sex	0.051
www		*F*(1, 32) = 0.406, *p* = 0.529		Main effect of treatment	0.094
xxx	PN4 cell body area, left hemisphere: male, female	*t*(56.06) = 0.0325, *p* = 0.0325	Normal	*t*-test	–311.4 to –14.03
yyy	PN4 cell body area, right hemisphere: male, female	*t*(48.57) = 2.088, *p* = 0.042	Normal	*t*-test	–353 to –6.739
zzz	PN4 number of dendrites, left hemisphere: male, female	*t*(73.5) = 0.9388, *p* = 0.3509	Normal	*t*-test	–1.024 to 0.368
aaaa	PN4 number of dendrites, right hemisphere: male, female	*t*(68.86) = 1.385, *p* = 0.1705	Normal	*t*-test	–0.2165 to 1.2
bbbb	PN4 number of nodes, left hemisphere: male, female	*t*(73.09) = 0.9446, *p* = 0.348	Normal	*t*-test	–1.935 to 0.6905
cccc	PN4 number of nodes, right hemisphere: male, female	*t*(71.89) = 0.643, *p* = 0.5223	Normal	*t*-test	–0.5776 to 1.128
dddd	PN4 spine density, left hemisphere: male, female	*t*(72.17) = 0.2635, *p* = 0.7929	Normal	*t*-test	–0.01184 to 0.01545
eeee	PN4 spine density, right hemisphere: male, female	*t*(72.52) = 0.06693, *p* = 0.9468	Normal	*t*-test	–0.01593 to 0.01489
ffff	PN4 total dendrite length, left hemisphere: male, female	*t*(74) = 1.527, *p* = 0.1309	Normal	*t*-test	–64.47 to 8.518
gggg	PN4 total dendrite length, right hemisphere: male, female	*t*(72.09) = 0.2125, *p* = 0.8323	Normal	*t*-test	–28.12 to 34.83
hhhh	PN4 average dendrite length, left hemisphere: male, female	*t*(71.66) = 1.452, *p* = 0.1509	Normal	*t*-test	–14.13 to 2.221
iiii	PN4 average dendrite length, right hemisphere: male, female	*t*(71.1) = 0.82898, *p* = 0.4094	Normal	*t*-test	–11.26 to 4.641
PN26 factor analysis: sex differences in neuronal morphology					
jjjj	Factor 1, left hemisphere: male, female	*t*(40.31) = 0.4664, *p* = 0.6435	Normal	*t*-test	–0.4694 to 0.7511
kkkk	Factor 1, right hemisphere: male, female	*t*(28.31) = 3.454, *p* = 0.0018	Normal	*t*-test	–1.479 to –0.378
llll	Factor 2, left hemisphere: male, female	*t*(43.65) = 0.2945, *p* = 0.7698	Normal	*t*-test	–0.6725 to 0.5011
mmmm	Factor 2, right hemisphere: male, female	*t*(30.22) = 0.7176, *p* = 0.4785	Normal	*t*-test	–0.4041 to 0.842
nnnn	Factor 3, left hemisphere: male, female	*t*(42.96) = 0.9127, *p* = 0.3665	Normal	*t*-test	–0.3008 to 0.798
oooo	Factor 3, right hemisphere: male, female	*t*(46) = 1.431, *p* = 0.1592	Normal	*t*-test	–0.9151 to 0.1546
PN26 factor analysis: effects of specific and dual agonism on neuronal morphology					
pppp	Factor 1, left hemisphere: female vehicle, female ACEA, female GP1a	*F*(2, 60) = 0.533, *p* = 0.0589	Normal	ANOVA	0.134
qqqq	Factor 1, left hemisphere, female vehicle: female ACEA+GP1a	*t*(38.27) = 0.4245, *p* = 0.6735	Normal	*t*-test	–0.4879 to 0.747
rrrr	Factor 1, right hemisphere: female vehicle, female ACEA, female GP1a	*F*(2, 60) = 2.099, *p* = 0.132	Normal	ANOVA	0.414
ssss	Factor 1, right hemisphere: female vehicle, female ACEA+GP1a	*t*(37.95) = 1.639, *p* = 0.1094	Normal	*t*-test	–1.193 to 0.1255
tttt	Factor 2, left hemisphere: female vehicle, female ACEA, female GP1a	*F*(2, 60) = 2.576, *p* = 0.085	Normal	ANOVA	0.494
	left hemisphere: female vehicle, female ACEA, female GP1a				
uuuu	Factor 2, left hemisphere: female vehicle, female ACEA+GP1a	*t*(36.6) = 0.2349, *p* = 0.8156	Normal	*t*-test	–0.5315 to 0.6708
vvvv	Factor 2, right hemisphere: female vehicle, female ACEA, female GP1a	*F*(2, 60) = 0.235, *p* = 0.791	Normal	ANOVA	0.085
wwww	Factor 2, right hemisphere: female vehicle, female ACEA+GP1a	*t*(39.38) = 1.888, *p* = 0.0664	Normal	*t*-test	–0.03766 to 1.099
PN26 dual agonism masculinizes neuronal morphology					
xxxx	Factor 1, left hemisphere: male vehicle, female vehicle, female ACEA+GP1a	*F*(2, 72) = 0.257, *p* = 0.774	Normal	ANOVA	0.089
yyyy	Factor 1, right hemisphere: male vehicle, female vehicle, female ACEA+GP1a	*F*(2, 72) = 5.689, *p* = 0.005	Normal	ANOVA	0.849
zzzz	Factor 1, right hemisphere: male vehicle, female vehicle	*p* = 0.008		Tukey's Post-hoc	–1.3958 to –0.1738
aaaaa	Factor 1, right hemisphere: female vehicle, female ACEA+GP1a	*p* = 0.012		Tukey’s *post hoc*	0.1473 to 1.4254
bbbbb	Factor 1, right hemisphere: male vehicle, female ACEA+GP1a	*p* = 1.000		Tukey’s *post hoc*	–0.5597 to 0.5628
ccccc	Factor 2, left hemisphere: male vehicle, female vehicle, female ACEA+GP1a	*F*(2, 72) = 0.956, *p* = 0.775	Normal	ANOVA	0.089
ddddd	Factor 2, right hemisphere: male vehicle, female vehicle, female ACEA+GP1a	*F*(2, 72) = 1.991, *p* = 0.144	Normal	ANOVA	0.398

## Results

### Effects of select CB1R or CB2R agonism or antagonism during early development on juvenile rough-and-tumble play behavior

**Fig. 2.**
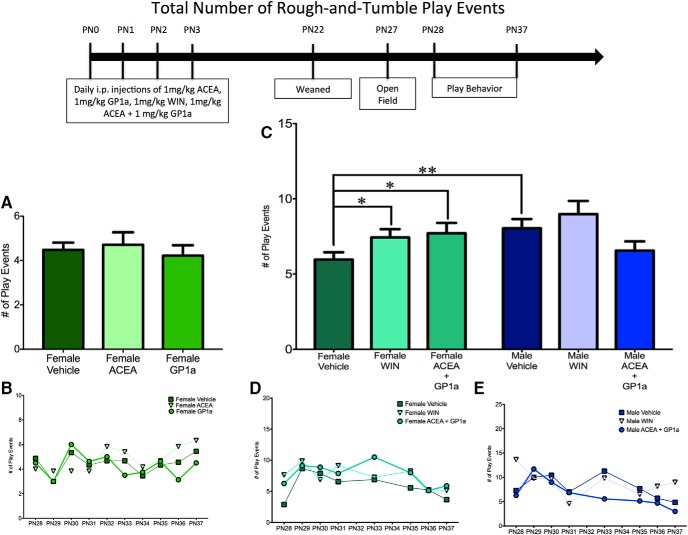
Activation of both CB1R and CB2R is necessary to masculinize the total frequency of female play behavior. ***A***, ***B***, Administration of ACEA (a CB1-specific agonist) or GP1a (a CB2-specific agonist) to neonatal (PN0–3) females did not alter the total frequency of juvenile rough-and-tumble play behavior (total of all pouncing, pinning, and boxing events from PN28-37). ***C–E***, Neonatal administration of WIN55,212-2 (WIN, a nonspecific endocannabinoid receptor agonist) or coadministration of ACEA and GP1a increased the frequency of play by juvenile females to the level observed in males but did not cause a further increase in male play. ***B***, ***D***, ***E***, Insets show the average frequency of rough-and-tumble play events for each day of analysis. *p < 0.05, **p < 0.01, *n* = 6–9.

**Fig. 3.**
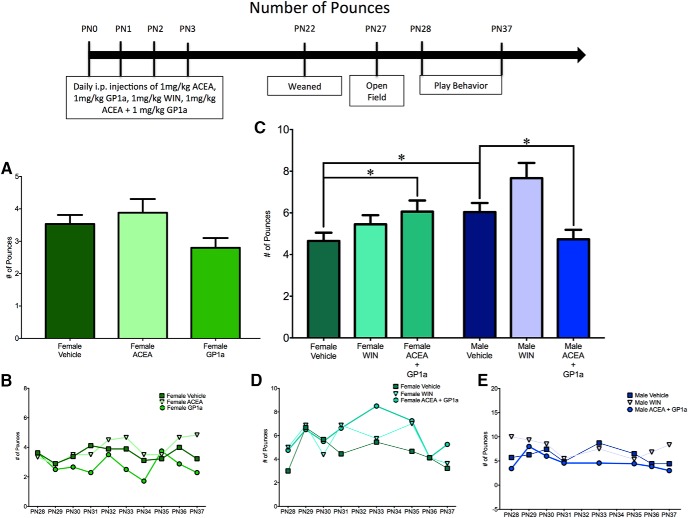
Activation of both CB1R and CB2R is necessary to masculinize female pouncing behavior. ***A***, ***B***, Administration of ACEA, GP1a, or WIN to neonatal (PN0–3) females did not alter the frequency of pouncing events. ***C–E***, Neonatal coadministration of ACEA and GP1a increased female pouncing and decreased male pouncing. Frequency of pouncing is an average over all days of analysis with the average for each individual day shown in the insets (***B***, ***D***, ***E***). *p < 0.05, *n* = 6–9.

**Fig. 4.**
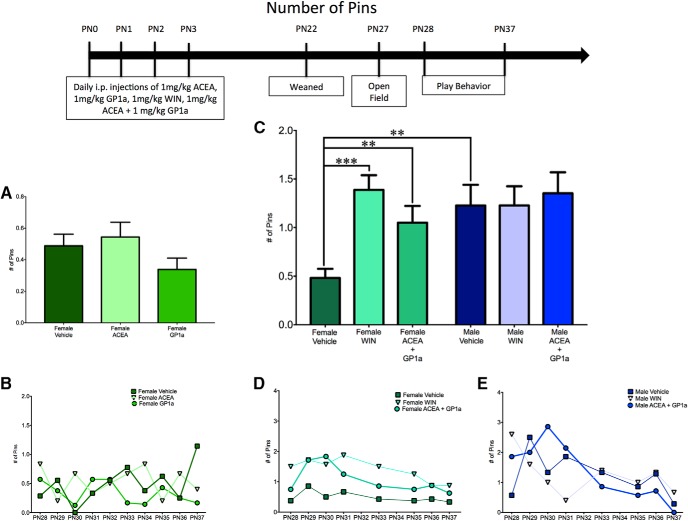
Activation of both CB1R and CB2R is necessary to masculinize female pinning behavior. ***A***, ***B***, Administration of ACEA or GP1a to neonatal (PN0–3) females did not alter the frequency of pinning events. ***C–E***, Neonatal coadministration of ACEA and GP1a or treatment with WIN increased the frequency of pinning by females to the level observed in males and had no effect on male pinning behavior. Frequency of pinning is an average over all days of analysis with the average for each individual day shown in the insets (***B***, ***D***, ***E***). **p < 0.01, ***p < 0.001, *n* = 6–9.

To determine whether the increase in female juvenile rough-and-tumble play behavior that was observed after neonatal treatment with WIN55,212-2 was due to signaling through the CB1R or CB2R, female pups (PN0–3) were treated daily with ACEA (a CB1R-specific agonist) or GP1a (a CB2R-specific agonist). The total number of play events in ACEA- and GP1a-treated females was comparable to that of vehicle-treated females^a^ (Fig. 2*A*, *B*). A breakdown of play into initiation (number of pounces) or response (number of pins) also demonstrated no effects of treatment^b,c^ (Figs. 3*A*, *B* and 4*A*, *B*). Open-field behavior was assessed to determine whether there were changes in locomotor (gridlines crossed) or anxiety-like (time spent in the center zone) behavior that could impact social play behavior. There was no effect of treatment with ACEA or GP1a on the number of gridlines crossed^d^ or time spent in the center zone^e^ (data not shown).

**Fig. 5.**
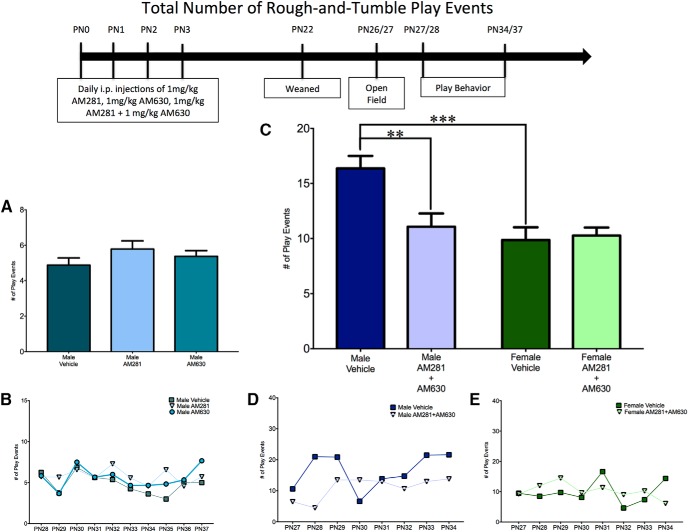
Combined antagonism of CB1R and CB2R is necessary to feminize the total frequency of male play behavior. ***A***, ***B***, Administration of AM281 (a CB1-specific antagonist) or AM630 (a CB2-specific antagonist) to neonatal males (PN0–3) did not alter the total frequency of rough-and-tumble play events. ***C–E***, Coadministration of AM281 and AM630 decreased the total frequency of rough-and-tumble play events by males to the level observed in females and had no effect on female play. Frequency of play is an average over all days of analysis, with the average for each individual day shown in the insets (***B***, ***D***, ***E***). **p < 0.01, ***p < 0.001, *n* = 6–10.

**Fig. 6.**
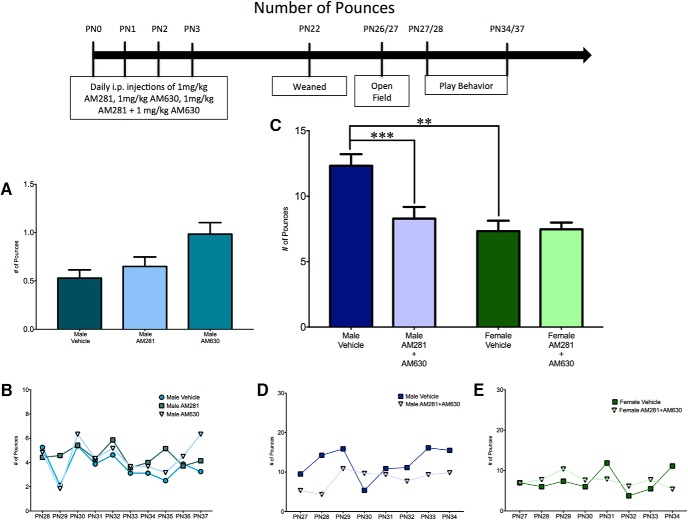
Combined antagonism of CB1R and CB2R is necessary to feminize pouncing behavior by males. ***A***, ***B***, Administration of AM281 or AM630 to neonatal males (PN0–3) did not alter the frequency of pouncing behavior. ***C–E***, Coadministration of AM281 and AM630 decreased pouncing behavior by males to the level observed in females and had no effect on female play. Frequency of pouncing is an average over all days of analysis, with the average for each individual day shown in the insets (***B***, ***D***, ***E***). **p < 0.01, ***p < 0.001, *n* = 6–10.

**Fig. 7.**
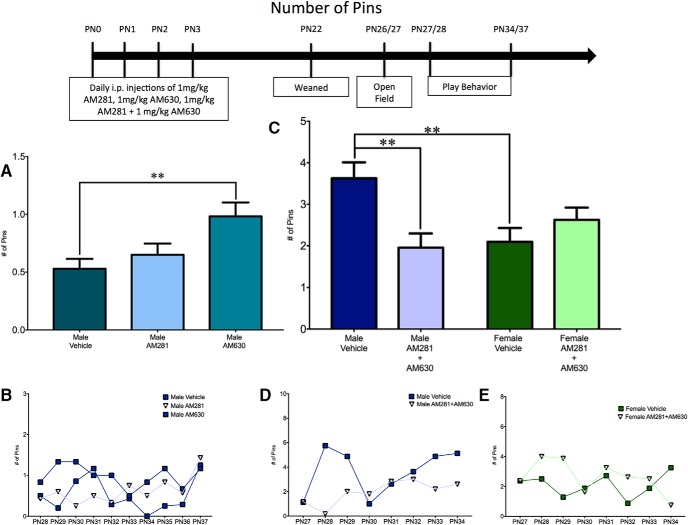
Combined antagonism of CB1R and CB2R is necessary to feminize pinning behavior by males. ***A***, ***B***, Administration of AM281 to neonatal males (PN0–3) did not alter the frequency of pinning behavior, whereas treatment with AM630 caused an increase in pinning. ***C–E***, Coadministration of AM281 and AM630 decreased pinning behavior by males to the level observed in females and had no effect on female play. Frequency of pinning is an average over all days of analysis, with the average for each individual day shown in the insets (***B***, ***D***, ***E***). **p < 0.01, *n* = 6–10.

Neonatal treatment with the pan-CBR agonist WIN55,212-2 does not effect play in juvenile males (Krebs-Kraft et al., 2010). Therefore we sought to determine whether antagonism of the CB1R and CB2R would decrease male play behavior. Neonatal male pups (PN0–3) were treated daily with AM251 (a CB1R-specific antagonist/inverse agonist) or AM630 (a CB2R-specific antagonist/inverse agonist). Treatment with AM281 or AM630 did not alter the total number of play events^f^ (Fig. 5*A*, *B*) or the number of pounces^g^ (Fig. 6*A*, *B*). However, CB2R antagonism increased the number of pins^h,j^ (Fig. 7*A*, *B*). Neither AM281 nor AM630 altered the number of gridlines crossed^k^ or the time spent in the center of an open field^l^ (data not shown).

#### Effects of combined CB1R or CB2R receptor agonism during early development on juvenile rough-and-tumble play behavior

Because of the surprising lack of changes to juvenile social play behavior after either specific agonism or antagonism of CB1R and CB2R, we administered a combination of ACEA and GP1a or WIN55,212-2 to neonatal (PN0–3) males and females to determine whether the combination of specific agonists would recapitulate the previous findings on the effect of treatment with WIN55,212-2. When assessed for the total number of juvenile rough-and-tumble play events, there was a significant interaction between sex and drug treatment^m^, with a main effect of sex^n^, but no main effect of treatment^0^ (Fig. 2*C–E*).

*A priori post hoc t*-tests were used to address our hypothesis that males would have a higher total number of play events than females. Neonatal treatment with WIN55,212-2 and ACEA+GP1a were predicted to increase female play to the level observed in males, and neither WIN55,212-2 nor ACEA+GP1a was predicted to alter male play. As expected, vehicle-treated males played more than vehicle-treated females^p^ (Fig. 2*C–E*; see Movie 1 and Movie 2 for a comparison of typical male and female rough-and-tumble play behavior, respectively). Neonatal treatment of females with both WIN55,212-2 and ACEA+GP1a increased the number of play events relative to control females^q,r^ (Fig. 2*C*, *D*). WIN55,212-2- and ACEA+GP1a-treated females and males all demonstrated a number of play events comparable to that of vehicle-treated males^s,t,u,v^ (Fig. 2*C–E*).

Movie 1. Representative example of male juvenile social play behavior. Two juvenile males (PN28) were allowed to acclimate to the chamber for 2 minutes, after which video recording was initiated. Instances of pouncing, pinning, and boxing were recorded from time 0 to 10:00. 10.1523/ENEURO.0344-16.2017.video.1

Movie 2. Representative example of female juvenile social play behavior. Two juvenile females (PN28) were allowed to acclimate to the chamber for 2 minutes, after which video recording was initiated. Instances of pouncing, pinning, and boxing were recorded from time 0 to 10:00. 10.1523/ENEURO.0344-16.2017.video.2

To determine the effects of neonatal treatment with WIN55,212-2 and ACEA+GP1a on initiation of play and response to play initiation, the total number of play events was split into pounces and pins. There was a significant sex × treatment interaction^w^ with a main effect of treatment^y^, but no main effect of sex^x^ for pouncing (Fig. 3*C–E*) and a significant sex × treatment^gg^ interaction with main effects of both sex^hh^ and treatment^ii^ for pinning (Fig. 4*C–E*).

Control males had higher levels of both pouncing^z^ and pinning^jj^ relative to females (Figs. 3*C–E* and 4*C–E*). Neonatal treatment with WIN55,212-2 increased pinning^kk^ in females, but did not alter pouncing^aa^, whereas treatment with ACEA+GP1a increased both pouncing^bb^ and pinning^ll^ (Figs. 3*C*, *D* and 4*C*, *D*). Both pouncing^cc,dd^ and pinning^mm,nn^ behavior of WIN55,212-2- and ACEA+GP1a-treated females was indistinguishable from that of vehicle-treated males (Figs. 3*C–E* and 4*C–E*). Although there were no changes in total play in males after neonatal treatment with WIN55,212-2 and ACEA+GP1a, there was a decrease in pouncing in ACEA+GP1a-treated males^ff^ (Fig. 3*C*, *E*). During the open field test neither prior drug treatment (ACEA+GP1a or WIN55,212-2) nor the sex of the animal influenced the number of gridlines crossed^qq,rr,ss^ or time spent in the center zone^tt,uu,vv^ of the open field (data not shown).

#### Effects of combined CB1R or CB2R antagonism during early development on juvenile rough-and-tumble play behavior

After determining that only combined agonism of CB1R and CB2R was sufficient to masculinize social play by females, we hypothesized that combined antagonism of CB1R and CB2R would dysmasculinize social play behavior in males. Additionally, based on the finding that combined agonism of CB1R and CB2R did not alter juvenile male behavior, we hypothesized that combined antagonism of CB1R and CB2R would have no effect on juvenile female play behavior. Neonatal (PN0–3) males and females were treated with AM281 and AM630. For total numbers of juvenile rough-and-tumble play events, there was a significant sex by treatment interaction^ww^, with a main effect of sex^xx^ and treatment^yy^ (Fig. 5*C–E*). As observed previously, vehicle-treated males played more than vehicle-treated females^zz^ (Fig. 5*C–E*). In support of our hypothesis, AM281+AM60-treated males played less than vehicle-treated males^aaa^ and were analogous to vehicle-treated females^bbb^ (Fig. 5*C–E*). As predicted, AM281+AM630-treated females were equivalent to vehicle-treated females^ccc^ (Fig. 5*C*, *E*).

A breakdown of total play into pouncing and pinning resulted in an interaction between sex and treatment for both pouncing^ddd^ and pinning^kkk^, as well as main effects of sex and treatment for pouncing^eee,fff^ but not pinning^lll,mmm^ (Figs. 6*C–E* and 7*C–E*). *A priori post hoc t*-test confirmed our hypotheses that juvenile males pounced^ggg^ and pinned more than females^nnn^, but males treated neonatally with AM281+AM630 pounced and pinned less than control males^hhh,000^ and were comparable to control females^iii,ppp^ (Figs. 6*C–E* and 7*C–E*). Neonatal treatment with AM281+AM630 did not alter female pouncing^jjj^ or pinning^qqq^ behaviors (Figs. 6*C*, *E* and 7*C*, *E*). Treatment with AM281+AM630 in males or females did not alter the number of gridlines crossed^rrr,sss,ttt^ or the amount of time spent in the center zone^uuu,vvv,www^ of the open field on PN26 (data not shown).

### Sex differences in neuronal morphology in the neonatal medial amygdala

**Fig. 8.**
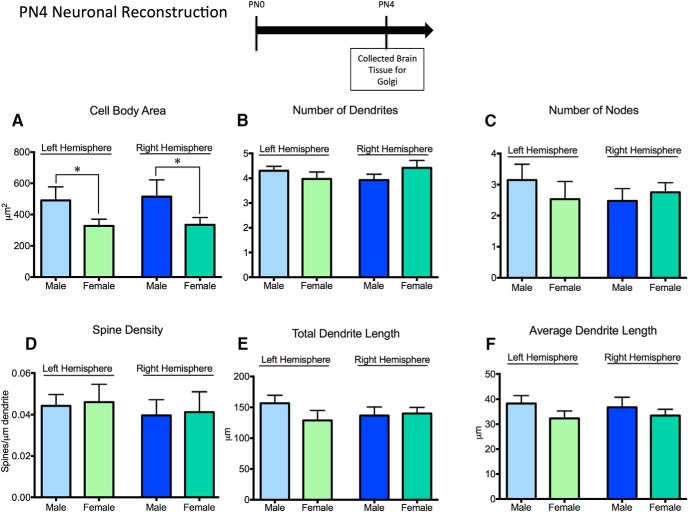
Few sex differences are found in neurons in the neonatal medial amygdala. 3D reconstruction of PN4 Golgi-impregnated neurons in the medial amygdala revealed sex differences in the cell body area in both hemispheres (***A***), but not in number of dendrites (***B***), number of nodes (***C***), spine density (***D***), total dendritic length (***E***), or average dendritic length (***F***). *p < 0.05, *n* = 10 individuals per group, with six neurons per hemisphere reconstructed for each individual.

We first investigated neuronal morphology at PN4 to determine whether sex differences were present during the time of our manipulation of the endocannabinoid system, which also corresponds with the sensitive period for sexual differentiation of neuronal circuitry. In the preoptic area, ventromedial nucleus, and arcuate nucleus of the hypothalamus, sex differences in neuronal morphology are already present at this age (Mong et al., 1999; Amateau and McCarthy, 2004). 3D reconstruction of Golgi-impregnated neurons in the medial amygdala indicated that males have larger cell bodies in both the right and left hemispheres^xxx, yyy^ relative to females, but there were no sex differences in numbers of dendrites^zzz, aaaa^ or nodes^bbbb, cccc^, spine density^dddd, eeee^, or total^ffff, gggg^ and average^hhhh, iiii^ dendrite length (Fig. 8).

### Sex differences in neuronal morphology in the juvenile medial amygdala

We next investigated sex differences in neuronal morphology at PN26. Cell body area, number of dendrites, number of nodes, spine density, average dendritic length, and total dendritic length were analyzed by factor analysis to identify patterns in how these parameters contributed to differences in neuronal morphology. Factor analysis identified three factors, together accounting for 73.9% of total variance, that were sufficient to explain the data. The first factor, which accounted for 34% of the total variance and was named Neuronal Complexity, contained positive loadings for number of nodes, total dendritic length, and average dendritic length. The second factor, which accounted for 23.1% of the total variance and was named Dendritic Field, contained positive loadings for the number of dendrites and total dendritic length and a negative loading for average dendritic length. The third factor, which accounted for 16.8% of the total variance and was named Spine Density, contained a positive loading for a single parameter, spine density (Fig. 10*A*).

**Fig. 9.**
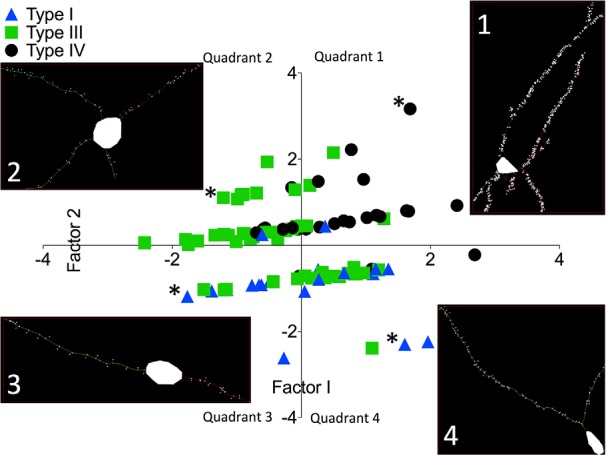
Factor analysis separates neurons into groups based on observable differences in neuronal morphology. 3D reconstruction of male and female PN26 Golgi-impregnated neurons in the medial amygdala, separation of the neurons into classes based on cell body shape, spine frequency, or numbers of dendrites, and graphing by factor scores for Factor 1 (Dendritic Complexity) vs. Factor 2 (Dendritic Field) shows how classes of neurons cluster together within specific quadrants. Type I neurons (blue triangles) had spindle-shaped cell bodies, 1–3 primary dendrites and clustered within quadrants 3 and 4. Type III neurons (green squares) contained ovoid cell body, 2–5 dendrites, moderate spine density, and clustered within quadrants 2 and 4. Type IV neurons (black circles) had ovoid cell bodies, 3–5 primary dendrites, numerous spines, and clustered within quadrant 1. In each quadrant, tracing for a representative neuron is shown with the corresponding data point indicated with an asterisk.

To determine how these factors corresponded to visual observations of differences in neuronal morphology, neurons from males and females were separated into three different classifications based on the classes used by Nishizuka et al. (1989). Type I neurons were defined based on the presence of a spindle-shaped cell body and one to three primary dendrites with numerous spines branching into a cylindrical field. We did not find any neurons belonging to the Type II class that were described as almost entirely lacking dendritic spines (Nishizuka et al., 1989). Type III neurons had ovoid cell bodies and two to five dendrites with some spines. Type IV neurons had three to five dendrites and were distinguished from Type III neurons based on their high spine density. Data points corresponding to individual neurons were color- and shape-coded based on their classification as Type I, III, or IV and graphed according to their factor scores for Dendritic Complexity and Dendritic Field (Fig. 9). The resulting scatter plot shows a clear linear separation of data points along the *y*-axis, corresponding to the scores for Dendritic Field. The grouping of points along this axis was attributed to the fact that all neurons must have at least one dendrite, making it impossible for a data point to fall at a position that would correspond to zero dendrites or fractions of dendrites. We chose a representative tracing of a neuron for each quadrant of the graph to illustrate that neurons with high factor scores for Dendritic Complexity and Dendritic Field (quadrant 1) were the most complex neurons, with several long dendrites and multiple branch points; neurons with high scores for Dendritic Complexity and low scores for Dendritic Field (quadrant 4) had few long dendrites with moderate branching; neurons with a low Dendritic Complexity score and a high Dendritic Field score (quadrant 3) had several dendrites that were relatively short with minimal branching; and neurons with low Factor 1 and Factor 2 scores (quadrant 2) had few short dendrites with minimal branching (Fig. 9). Neurons that we classified as Type I grouped together within quadrants 3 and 4 on the graph, Type III neurons grouped within quadrants 2 and 4 on the graph, and Type IV neurons grouped mostly within quadrant 1 (Fig. 9).

**Fig. 10.**
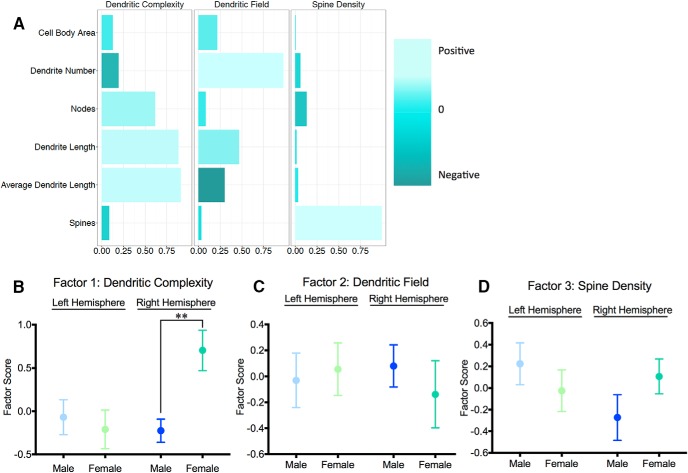
Juvenile females have greater dendritic complexity in the right hemisphere relative to males. 3D reconstruction of male and female PN26 Golgi-impregnated neurons in the medial amygdala and factor analysis of cell body area, number of dendrites, number of nodes, total dendritic length, average dendritic length, and spine density (***A***) identified three factors (Dendritic Complexity, Dendritic Field, and Spine Density) depicted with longer aqua (lighter) bars indicating positive loading of a parameter and teal (darker) indicating negative loading. Factor scores for neurons from vehicle males and females were plotted and compared for Dendritic Complexity (***B***), Dendritic Field (***C***), and Spine Density (***D***). **p < 0.01, *n* = 3–5 individuals per group, with six neurons per hemisphere reconstructed for each individual.

To quantify sex differences in these three factors, we calculated and compared factor scores for neurons from females and males in both the right and left hemisphere. For Dendritic Complexity, males and females were not different in the left hemisphere^jjjj^; however, females had higher factor scores in the right hemisphere^kkkk^, suggesting that females have greater dendritic complexity in the right hemisphere relative to males (Fig. 10*B*). For Dendritic Field and Spine Density, males and females did not differ significantly in either hemisphere^llll,mmmm,nnnn,oooo^ (Fig. 10*C*, *D*).

### Comparison of neonatal select or dual agonism of CB1R and CB2R on neuronal morphology in the juvenile medial amygdala

**Fig. 11.**
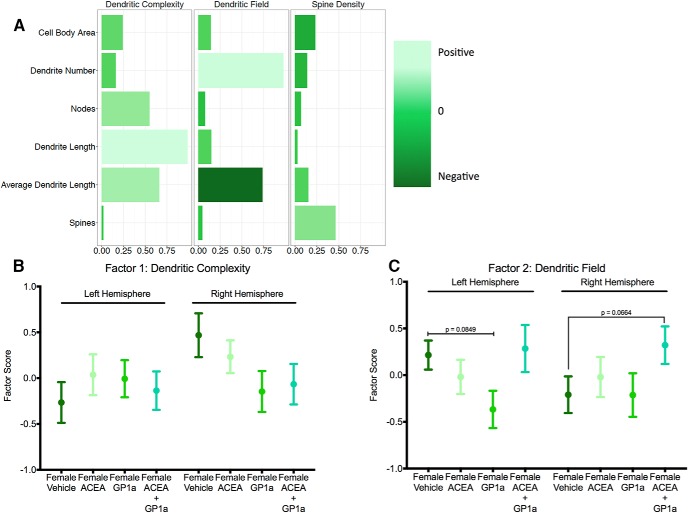
Combined neonatal agonism of CB1R and CB2R uniquely shifted juvenile female dendritic field relative to selective agonism of either receptor. 3D reconstruction of female PN26 Golgi-impregnated neurons in the medial amygdala from animals treated neonatally with ACEA, GP1a, ACEA+GP1a, or vehicle and factor analysis of cell body area, number of dendrites, number of nodes, total dendritic length, average dendritic length, and spine density (***A***) identified three factors (Dendritic Complexity, Dendritic Field, and Spine Density) depicted with longer light green bars indicating positive loading of a parameter and dark green indicating negative loading. Factor scores for neurons from ACEA, GP1a, ACEA+GP1a, and vehicle females were plotted and compared for Dendritic Complexity (***B***) and Dendritic Field (***C***). Exact *p* values are indicated, *n* = 3–5 individuals per group, with six neurons per hemisphere reconstructed for each individual.

Factor analysis accounted for 60.7% of the total variance (Fig. 11*A*). Dendritic Complexity accounted for 29.9% of the variance, whereas Dendritic Field explained 25.6% of the total variance. Spine Density accounted for 5.2% of the total variance and was excluded from further analysis. We calculated factor scores for neurons from the right and left hemisphere from each treatment group. For Dendritic Complexity, there was no effect of specific or dual agonism in either hemisphere^pppp, qqqq, rrrr, ssss^ (Fig. 11*B*). For Dendritic Field in the left hemisphere, there was a slight trend toward a significant effect of select agonism^tttt^ and no effect of dual agonism^uuuu^, whereas in the right hemisphere there was no effect of select agonism^vvvv^ and a trend toward a significant effect of dual agonism^wwww^ (Fig. 11*C*). These trends in the data suggest a shift in morphology toward a decreased dendritic field size in the left hemisphere after neonatal specific agonism and a shift toward a larger dendritic field in the right hemisphere after neonatal dual agonism.

### Neonatal dual agonism of CB1R and CB2R masculinizes neuronal morphology in the juvenile medial amygdala

**Fig. 12.**
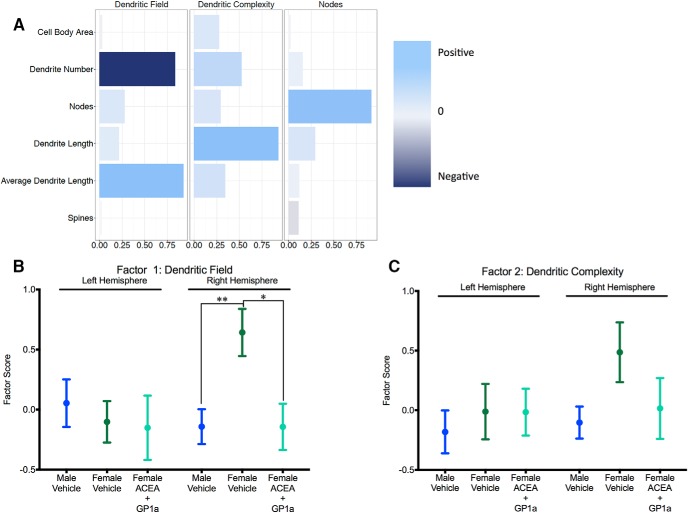
Neonatal agonism of CB1R and CB2R masculinize juvenile female dendritic field in the right hemisphere. 3D reconstruction of male and female PN26 Golgi impregnated neurons in the medial amygdala from animals treated neonatally with ACEA+GP1a or vehicle and factor analysis of cell body area, number of dendrites, number of nodes, total dendritic length, average dendritic length, and spine density (***A***) identified three factors (Dendritic Field, Dendritic Complexity, and Nodes) depicted with longer light blue bars indicating positive loading of a parameter and dark navy blue indicating negative loading. Factor scores for neurons from vehicle male and females and ACEA+GP1a-treated females were plotted and compared for Dendritic Field (***B***) and Dendritic Complexity (***C***). *p < 0.05, **p < 0.01, *n* = 3–5 individuals per group, with 6 neurons per hemisphere reconstructed for each individual.

Factor analysis identified three factors that accounted for 67.8% of the total variance. This included Dendritic Field and Dendritic Complexity as before, but a new factor was added named Nodes, which contained positive loading for number of nodes but was excluded from further analysis (Fig. 12*A*). Dendritic Field accounted for 28% of the variance, and Dendritic Complexity accounted for 23.7%. For Dendritic Field, there were no differences in the left hemisphere^xxxx^, but there was a significant effect in the right hemisphere^yyyy^ where males had lower factor scores relative to females^zzzz^, and females treated neonatally with ACEA+GP1 had lower factor scores relative to control females^aaaaa^ and were comparable to control males^bbbbb^. For Dendritic Field, there were no differences in the left hemisphere^ccccc^, and in the right hemisphere the factor scores showed trends similar to those for Dendritic Complexity, with control females loading higher than control males and ACEA+GP1a-treated females; however, these differences were not significant^ddddd^.

## Discussion

Social play behavior is engaged in by juveniles but is programmed developmentally during the sensitive period for sexual differentiation of the brain, resulting in higher levels of rough-and-tumble play in males. The neurobiological mechanisms by which this sex difference is established remain poorly understood. We here build on a previous observation of a role for the endocannabinoid system in masculinizing the neural circuits of play. We found that both CB1 and CB2 receptors are essential to masculinization of juvenile rough-and-tumble play behavior. Combined administration of CB1R and CB2R agonists to females during the sensitive period increased their play to the level of males, but the same treatment in males caused no further increase in play. Conversely, combined treatment with CB1R and CB2R antagonists decreased play in males to the level of females, without further decreasing play by females. The increase in female play behavior with CB1R and CB2R agonism and the decrease in male play behavior with CB1R and CB2R antagonism brought both pouncing and pinning components of play to the levels observed in the opposite sex, demonstrating full masculinization or feminization of the style of play following developmental manipulation of the endocannabinoid system. These data reveal cooperation between the CB1R and CB2R during development to direct development of the neural circuitry regulating juvenile play behavior.

For manipulations that resulted in feminization and masculinization of play, breakdown of play into the components of pouncing and pinning revealed some unexpected effects. In animals that had been treated neonatally with dual agonists, we observed an increase in female pouncing behavior, but saw a decrease in males. This opposite action between males and females could be the result of sex differences in the endocannabinoid system at the level of receptor expression or cell type localization of receptors. Additionally, our manipulations may have caused sex-specific compensatory mechanisms, such as desensitization of receptors or upregulation of endocannabinoids and their receptors. The existence of such compensatory mechanisms is supported by the observation that agonism did not produce a further increase in male play behavior and antagonism did not result in decreased female play behavior. It is unlikely that the lack of effect in the opposite sex was due to ceiling or basement levels of play, since other manipulations, such as introduction of a novel play partner or social isolation, can decrease female play and increase male and female play, respectively (for example, Beatty et al., 1982; Argue and McCarthy, 2015a). Because dual antagonism of the receptors during development decreases pinning in juvenile males, it was surprising to find increased pinning with selective CB2R antagonism in males. Pinning requires action on the part of both animals involved in the play bout: the pinner needs to be standing on top of his/her opponent which usually occurs after a pounce, and the pinnee needs to rotate to a supine position. Because this increase in pinning occurred without a corresponding increase in pouncing, it is possible that it was the result of one or more of the other animals in the group rotating to supine without being first pounced on. Such rotation to supine in the absence of a pounce is a rare event; however, the likelihood of this occurring increases when there is an increase in the distance at which defense is initiated (Himmler et al., 2013, 2016). This suggests a difference in perception of the AM630-treated male as a potential play partner causing others to rotate as he approaches rather than waiting for the pounce. Further paired play studies will be needed to determine whether the difference in pinning was the result of interaction with a specific play partner. In two cases, neonatal treatment with WIN55,212-2 produced an effect different from that of ACEA+GP1a. WIN55,212-2 treatment induced an increased total frequency of play by females that was due mostly to an effect of pinning rather than a combination of pinning and pouncing as was observed with ACEA+GP1a treatment, and the same treatment produced a decrease in male pouncing that was not observed with WIN55,212-2. We expected WIN55,212-2 and ACEA+GP1a to have similar effects. A possible reason for these discrepancies is the CB2R binding bias of WIN55,212-2, rather than more equal distribution between CB1R and CB2R that is achieved with ACEA+GP1a. WIN55,212-2 has a *K_i_* of 62.3 nm at CB1R and 3.3 nm at CB2R, whereas ACEA has a *K_i_* of 1.4 nm for CB1R and GP1a has a *K_i_* of 0.037 nm for CB2R. There is also the possibility that once these agonists bind to their receptor, different downstream signaling pathways are activated, as seen by others (Delgado-Peraza et al., 2016; Dhopeshwarkar and Mackie, 2016).

Emphasis in research on the role of endocannabinoids in CNS development has been on CB1R, which is considered the most abundant G-protein–coupled receptor in the brain and is found at high to moderate levels throughout (Herkenham et al., 1990). In the adult brain CB1R is predominantly on presynaptic terminals of glutamatergic and GABA-ergic neurons, with lower expression on astrocytes and microglia (Herkenham et al., 1990; Galiègue et al., 1995; Elphick and Egertová, 2001; Stella, 2010). There is a developmental switch in CB1R distribution such that it begins in the white matter and then shifts to being more abundant in the gray matter as the brain matures (Berrendero et al., 1998).

The role of CB2R in brain development is less explored and at times contradictory. Classically, CB2R is referred to as the peripheral endocannabinoid receptor (Munro et al., 1993). In recent years, however, there is growing evidence of the presence and abundance of CB2R in the CNS. Both CB1R and CB2R messenger RNAs can be detected in the brain of the developing fetus as early as 8 d of gestation in the laboratory rat (Buckley et al., 1998). Initial reports limited CB2R expression to microglia, the immune cells of the brain, or astrocytes, with expression on neurons still reserved only for CB1R (Núñez et al., 2004; Maresz et al., 2005; Benito et al., 2008; Racz et al., 2008a, 2008b). A few studies reported CB2R abundantly expressed on neurons throughout the brain, but full validation was lacking (Gong et al., 2006; Onaivi et al., 2006, 2008). In the ventral tegmental area of adult mice, CB2R receptors are immunohistochemically identified on dopaminergic neurons, where they modulate neuronal activity and dopamine-mediated cocaine self-administration, but these effects are absent in CB2R knockout mice (Zhang et al., 2014). CB2R is also localized to CA3 and CA2 pyramidal cells, where their activation leads to prolonged hyperpolarization (Stempel et al., 2016). Important to the current findings, all of these studies on CB2R localization were conducted in adults. Exceedingly little is known regarding the expression of this receptor in the developing brain.

The endocannabinoid system is critical for many neurodevelopmental processes, including neural progenitor proliferation and survival (Rueda et al., 2002; Aguado et al., 2005; Jiang et al., 2005; Palazuelos et al., 2006, 2012; Goncalves et al., 2008; Mulder et al., 2008; Rubio-Araiz et al., 2008; Galve-Roperh et al., 2013; Díaz-Alonso et al., 2015; Rivera et al., 2015), differentiation (Aguado et al., 2006; Gomez et al., 2010; Soltys et al., 2010; Galve-Roperh et al., 2013; Sun et al., 2013), and axonal growth and guidance (Williams et al., 2003; Berghuis et al., 2007; Mulder et al., 2008; Wu et al., 2010; Tortoriello et al., 2014). Potential endpoints for the modulation of social play behavior include cell proliferation or neuronal morphology in the medial amygdala. Krebs-Kraft et al. (2010) found a sex difference in cell proliferation that was due, in part, to higher numbers of newly proliferated astrocytes in the female neonatal medial amygdala, which is reversed by administration of WIN55,212-2 during the same period that WIN55,212-2 masculinizes female social play behavior. Combined treatment with WIN55,212-2 and antagonisms for CB1R or CB2R suggested, but did not confirm, that the effects were mediated by CB2R (Krebs-Kraft et al., 2010).

There is a sex difference in neuronal morphology in the medial amygdala of prepubertal rats, and sex differences in the number of neurons in the medial amygdala in adult animals is thought to be organized during early development (Cooke et al., 2007; Morris et al., 2008). These endpoints could work together, such that altering the number and type (i.e., neuron or astrocyte) during development could result in changed neuronal morphology. Additionally, there is a sex difference in the density of dendritic spines on neurons in the medial amygdala of adult rats, with males having greater spine frequency relative to females (Nishizuka et al., 1989). We did not find these same sex differences in the neonatal medial amygdala. It is possible that sex differences are present at this young age but are only noticeable once the neurons have extended further, or that our manipulations to the endocannabinoid system acted directly on a different aspect of neurodevelopment that produced later changes in neuronal morphology. In the juvenile medial amygdala, our factor analysis revealed sex differences in a factor comprised of measures of dendritic complexity (number of nodes, total dendritic length, and average dendritic length) in the right hemisphere. Neurons from females had higher factor scores for this factor, indicating that female neurons in the right hemisphere are more complex than males. We did not observe the previously reported sex differences, where males have greater dendritic branching and length in the left hemisphere and greater numbers of spines (Nishizuka et al., 1989; Cooke et al., 2007). Methodological differences between studies could account for some of the discrepancies, such as age of the animals, Golgi impregnation versus filling with biocytin, separation of the hemispheres, and classification of the neurons by general morphology. Our second factor analysis, designed to determine whether neonatal dual agonism of CB1R and CB2R affects neuronal morphology differently than select agonism of either receptor, demonstrated trends in the data that suggest a specific effect of simultaneous agonism that is not simply an addition of the effects observed when CB1R and CB2R are activated separately. Our third factor analysis determined that neonatal dual agonism of CB1R and CB2R was sufficient to masculinize dendritic field, which consisted of a negative association between the number of dendrites and the average dendritic length, in the right hemisphere. This analysis also suggested a slight trend toward masculinization of dendritic complexity (number of dendrites and total and averaged dendritic length) in the right hemisphere. Because our factor analysis of juvenile neuronal morphology was performed on a different cohort of animals from those that were used for the analysis of play behavior, a direct association between neuronal morphology and play behavior was not possible. However, there was a clear correlation between the requirement for activation of both CB1R and CB2R during the early neonatal period to masculinize juvenile female play behavior and the ability of this same treatment paradigm to shift neuronal morphology in the right hemisphere of the female medial amygdala closer to that of males.

In the current study, endocannabinoid receptor agonists and antagonists were given i.p.; therefore, we cannot rule out the potential of a peripheral mechanism of action. CB1R is lower in the periphery relative to the brain, and the opposite is true for CB2R (Galiègue et al., 1995). CB1R is mainly in the gastrointestinal tract, heart, liver, adipose tissue, lungs, adrenal glands, muscle, reproductive system, bone, and skin, whereas CB2R is associated with immune-related organs and cells, such as monocytes, macrophages, B and T cells, mast cells, keratinocytes, spleen, tonsils, thymus gland, and gastrointestinal tract (Izzo, 2004; Ständer et al., 2005; Matias and Di Marzo, 2006, 2007; Iannotti et al., 2014; Staiano et al., 2016). There is a precedent for communication between the peripheral immune system and the CNS. Under certain stress conditions, peripheral monocytes are recruited into the brain where they cause an increase in central inflammatory signaling (Weber et al., 2017). This process requires changes to blood–brain barrier permeability and a breakdown of astrocytic barriers (Weber et al., 2017). Manipulations to the endocannabinoid system could cause an increase in either pro- or anti-inflammatory signaling in peripheral immune components that then access the brain. Modulation of the endocannabinoid system may also directly induce changes to the blood–brain barrier (Engelhardt, 2003; Rom et al., 2013; Vendel and de Lange, 2014), thereby altering the inflammatory status of the brain, without acting on receptors located within the CNS. This peripheral mechanism of action is not necessarily mutually exclusive with a central mechanism of action. If we are to take the canonical classification of CB1R as central and CB2R as peripheral, then changes to CB2R signaling in the periphery may alter the peripheral immune system, which then communicates to the brain and alters CB1R signaling.

In summary, these data demonstrate sex-specific modulation of the developmental organization of juvenile rough-and-tumble play behavior via simultaneous signaling through both CB1 and CB2 receptors. Further investigation into how the endocannabinoid system interacts with sex steroid hormones and how CB1 and CB2 receptors act in cooperation to modulate acquisition of sexually differentiated play behavior will provide valuable insight into the functioning of the endocannabinoid system during early brain development and highlight potential ways in which cannabis use during pregnancy could have unintended effects on the developing fetus.

## Synthesis

Reviewing Editor: Cheryl Sisk, Michigan State University Decisions are customarily a result of the Reviewing Editor and the peer reviewers coming together and discussing their recommendations until a consensus is reached. When revisions are invited, a fact-based synthesis statement explaining their decision and outlining what is needed to prepare a revision will be listed below. The following reviewer(s) agreed to reveal their identity: Ismael Galve-Roperh, Sergio Pellis.

Two experts and I have reviewed your manuscript and agree that it reports interesting data that advance understanding of the role of endocannabinoids on the sexual differentiation of play behavior. To strengthen your paper, please consider the specific questions/comments below and revise your manuscript as appropriate. I also request that you double check your statistical results throughout, and make sure that the p values provided in the table accurately reflect the narrative provided in the results. Two inconsistencies were noted by reviewers: 1) Line 285-87 reports a significant interaction (m), but m in the stats table shows p=0.12; 2) Line 314 reports a treatment-dependent decrease in pouncing in males (pp) (fig 3c,e), but pp in stats table shows p=0.68. Finally, it would be helpful to include the figure captions in the body of your revised manuscript so that reviewers do not have to open the separate figure files to view the captions.

Line 92: A suitable reference to support and illustrate the point made by the first sentence would be Siviy, S. M. (2016). A brain motivated to play: Insights into the neurobiology of playfulness. Behaviour, 153, 819-844.

Lines 123-176: The first two experiments involved play encounters among 6 individuals of differing treatment and sex, whereas the third experiment involved using dyads of same sex and same treatment. The explanation offered in lines 168-176 provides a clear rationale as to why the dyad approach is advantageous for detecting sex and treatment effects. There is no rationale provided for the use of the six subject trials used for the first two experiments and indeed, the rationale for the use of dyads in the third experiment makes the choice of design in the first two experiments seem the weaker approach. Why wasn't the dyad paradigm used for all the experiments? The contagion effect could well have distorted the findings from the first two experiments. The methodology used needs to be thoroughly rationalized, with the possible pros and cons borne in mind when the findings are discussed.

Lines 178-214: A rationale needs to be provided to make clear the logic for why brains from both PN4 and PN26 rats were needed for reconstruction. Given that the animals were tested behaviorally in the juvenile period, why not just look at their brains for treatment effects at this age? The added value of the PN4 analysis needs to be provided. Also, given the many uses of independent groups of rats involved, it would be useful to state the total number of male and female rats used for the entire study up front in the Methods under “Animals” (line 111-121). This would save the reader trying to calculate overall animal usage from the separate experiments.

Lines 285-287: The ending sentence states that “When assessed for the total number of juvenile rough-and-tumble play events, there was a significant interaction between sex and drug treatment, with a main effect of sex, but no main effect of treatment”. However, in the subsequent paragraphs statistical results are given for some treatment effects on females. That is, specific comparisons are made without justification from an overall effect. If the ANOVA reported a significant interaction effect, which according to the statistical table, it did not, then this would be justified, otherwise a case needs to be made to justify the subsequent comparisons further.

Lines 312-314: The absence of a decrease in overall play in the treated males, but a decrease in pouncing, suggests that overall play may be sustained by an increased likelihood that treated males are responsive to nape contacts. That is, there may be a differential treatment-induced change on playful attack versus playful defense - consequently, the different receptors may have differential effects on different aspects of play fighting.

Lines 443-461: Please provide a possible explanation for why ECBr agonism, but not antagonism, in females masculinizes their social play, whereas the opposite is the case in males, i.e., antagonism reduces social play while agonism does not impact the behavior. For example, do you think there are ceiling or basement effects going on, and if so, are there possible mechanistic reasons why this would be the case?

Lines 462-494: Rotating to supine in the absence of a nape attack by a partner is rare (Himmler, S. M., Himmler, B. T., Stryjek, R., Modlińska, K., Pisula, W., & Pellis, S. M. (2016). Pinning in the play fighting of rats: A comparative perspective with some methodological recommendations. International Journal of Comparative Psychology, 29, 1-14), consequently, if a treatment from the present experiment enhanced such behavior, this would be an important finding and one indicating separate neural controls over communication during play compared to regulating combat actions. An alternative way to increase the likelihood of rolling to supine prior to contact is to increase the distance at which defense is initiated (see Himmler, B. T., Stryjek, R., Modlińska, K., Derksen, S. M., Pisula, W., & Pellis, S. M. (2013). How domestication modulates play behavior: A comparative analysis between wild rats and a laboratory strain of Rattus norvegicus. Journal of Comparative Psychology, 127, 453-464; Pellis et al., 1997, cited). That is, the defensive of the recipient is altered, not its propensity to solicit play. Indeed, some findings in the present paper (see comment above for lines 312-314) - in which playful pouncing can decrease in males even though overall play is sustained, suggesting a concomitant increase in rearing to box or in rolling to supine - would support such an alternative. Playful attack and playful defense show different age-related changes in males and females, with different patterns of hormonal organization involved in the sexes (see Pellis, 2002, cited). Therefore, it is possible that even if the CB receptors are involved in both males and females, their relative contributions to the sex differences in playful attack and playful defense may differ. This is certainly a possibility to be considered for future studies.

Lines 533-541: Please clarify how you think ECB/CB2R effects on newly proliferated astrocytes in the female medial amygdala (Krebs-Kraft et al) relate to effects (or lack thereof) of selective CB2R agonism/antagonism on play behavior.
